# Evaluation of the Properties of the DNA Methyltransferase from Aeropyrum pernix K1

**DOI:** 10.1128/Spectrum.00186-21

**Published:** 2021-09-29

**Authors:** Mao Hayashi, Keisuke Sugahara, Akira Yamamura, Yasuhiro Iida

**Affiliations:** a Department of Applied Chemistry and Bioscience, Graduate School of Engineering, Kanagawa Institute of Technology, Atsugi, Kanagawa, Japan; b Department of Applied Bioscience, Faculty of Applied Bioscience, Kanagawa Institute of Technology, Atsugi, Kanagawa, Japan; Temasek Life Sciences Laboratory

**Keywords:** *Aeropyrum pernix*, archaea, DNA methylation, DNA methyltransferase, epigenetics

## Abstract

Little is known regarding the DNA methyltransferases (MTases) in hyperthermophilic archaea. In this study, we focus on an MTase from Aeropyrum pernix K1, a hyperthermophilic archaeon that is found in hydrothermal vents and whose optimum growth temperature is 90°C to 95°C. From genomic sequence analysis, *A. pernix* K1 has been predicted to have a restriction-modification system (R-M system). The restriction endonuclease from *A. pernix* K1 (known as ApeKI from New England BioLabs Inc. [catalog code R06435]) has been described previously, but the properties of the MTase from *A. pernix* K1 (M.ApeKI) have not yet been clarified. Thus, we demonstrated the properties of M.ApeKI. In this study, M.ApeKI was expressed in Escherichia coli strain JM109 and affinity purified using its His tag. The recognition sequence of M.ApeKI was determined by methylation activity and bisulfite sequencing (BS-seq). High-performance liquid chromatography (HPLC) was used to detect the position of the methyl group in methylated cytosine. As a result, it was clarified that M.ApeKI adds the methyl group at the C-5 position of the second cytosine in 5′-GCWGC-3′. Moreover, we also determined that the MTase optimum temperature was over 70°C and that it is strongly tolerant to high temperatures. M.ApeKI is the first highly thermostable DNA (cytosine-5)-methyltransferase to be evaluated by experimental evidence.

**IMPORTANCE** In general, thermophilic bacteria with optimum growth temperatures over or equal to 60°C have been predicted to include only N^4^-methylcytosine or N^6^-methyladenine as methylated bases in their DNA, because 5-methylcytosine is susceptible to deamination by heat. However, from this study, *A. pernix* K1, with an optimum growth temperature at 95°C, was demonstrated to produce a DNA (cytosine-5)-methyltransferase. Thus, *A. pernix* K1 presumably has 5-methylcytosine in its DNA and may produce an original repair system for the expected C-to-T mutations. M.ApeKI was demonstrated to be tolerant to high temperatures; thus, we expect that M.ApeKI may be valuable for the development of a novel analysis system or epigenetic editing tool.

## INTRODUCTION

DNA methylation is an epigenetic modification that is found in eukaryotes, prokaryotes, and viruses (1). DNA methylation is involved in significant biological functions, such as X chromosome inactivation in females, genomic imprinting, and gene expression regulation in eukaryotic cells (2, 3). In prokaryotes, DNA methylation also has significant functions, including roles in restriction-modification (R-M) systems, indicating daughter strands for the mismatch repair system following DNA replication, and in cell cycle regulation (4, 5). Methyl groups are added by a DNA methyltransferase (MTase) from *S*-adenosylmethionine (SAM) as the donor molecule (6). MTases essentially have two types in mammals as follows: the maintenance MTases have a preference for hemimethylated DNA (7), and the *de novo* type MTases have no such preference (8). Conversely, this distinction is less clear in bacteria/archaea. In bacteria/archaea, N^6^-methyladenine (m6A), 5-methylcytosine (m5C), and N^4^-methylcytosine (m4C) have all been reported.

In prokaryotes, the functions of N^6^-methyladenine in the genomic DNA have been studied in DNA replication and repair, gene expression regulation, and immunity via the R-M system. (9). In eukaryotic cells, however, m6A had not been discovered until recently, when it was identified in Caenorhabditis elegans by methylated DNA immunoprecipitation sequencing (MeDIP-seq) (10). m6A has been identified in the DNA of many eukaryotes, including Drosophila melanogaster, Bombyx mori, Arabidopsis thaliana, Mus musculus, and Homo sapiens, and its function is suggested to be cell cycle control and gene expression regulation (11).

5-Methylcytosine has been widely studied in eukaryotic cells, where it acts in general terms as a transcription repressor. m5C is enriched in CpG islands in promoter regions, where DNA methylation inhibits gene expression due to the inhibition of transcription factor binding either directly or via mediation of methyl binding domain proteins that recruit histone deacetylase (HDAC) (12). Furthermore, m5C is related to development and differentiation (1). In Escherichia coli, DNA cytosine methylase (Dcm) is not part of an R-M system, and even in this mesophilic organism, the resulting m5C-to-T mutations are repaired by a “very short patch” repair system (13).

N^4^-methylcytosine is observed in thermophilic bacteria and archaea (14). The studies of Wang et al. and Ehrlich et al. showed that m5C is changed to thymine by demethylation under high temperatures (15, 16). The optimum growth temperature of thermophilic bacteria is over 60°C. Thus, thermophilic bacteria have been suggested to use m4C, which is resistant to heat, instead of m5C to minimize genome mutation (17).

In particular, m6A, m5C, and m4C, in the context of R-M systems, have been well studied in bacteria. An E. coli R-M system, for example, was reported as an immunity system around 70 years ago (18). The R-M system constitutes two factors as follows: restriction endonucleases (REases), which cleave-specific sequences, and MTases, which add methyl groups within the specific sequence. This system, among other roles, protects host cells from phage infection. The invasive phage DNA is cleaved by the REase, whereas the genomic DNA is protected by the cognate DNA MTase (19).

Although MTases have been studied in various biological species, little is known regarding thermostable MTases. The study of Watanabe et al. showed that M.PabI, an MTase from the hyperthermophilic archaeon Pyrococcus abyssi, had the highest optimum temperature among then-known DNA (adenine-6)-methyltransferases (20). M.PabI is part of an R-M system and recognizes the sequence 5′-GTAC-3′ (20). The optimum temperature of M.PabI is 85°C, and activity has been identified at temperatures higher than 95°C (it retains at least half of its activity after incubation at 95°C for 9 min) (20).

Conversely, a thermostable DNA (cytosine-5)-methyltransferase has not yet been reported. A thermostable DNA cytosine methyltransferase was reported by Nölling and de Vos (21). In the article, MTase from Methanobacterium thermoformicicum THF (M.MthTI) was predicted to be DNA (cytosine-5)-methyltransferase just from amino acid alignment, and there are no experiments demonstrating the position at which the cytosine was methylated by M.MthTI.

In this study, we focus on an MTase of the hyperthermophilic archaeon, Aeropyrum pernix K1, whose genome analysis is complete (22). According to the National Institute of Technology and Evaluation, the optimum growth temperature of *A. pernix* K1 is 90°C to 95°C. The enzymes of *A. pernix* K1 are all presumed to be thermostable. Thus, they have been used in biotechnology applications. For example, uracil-DNA glycosylase from *A. pernix* K1 is used in hot-start PCR (23). *A. pernix* K1 is predicted to have an R-M system. The REase of *A. pernix* K1 is already known as ApeKI and recognizes the sequence 5′-GC(A/T)GC-3′. In contrast, the MTase of *A. pernix* K1, which was designated as M.ApeKI (24) (UniProt accession number Q9YDP3) has only been evaluated by homology analysis (25). Watanabe et al. mention that not only the activity of M.ApeKI but also the expression of the enzyme has not yet been reported (20).

In this study, we investigated the properties of M.ApeKI. The recognition sequence of M.ApeKI was evaluated by methylation activity and bisulfite sequencing (BS-seq), and we used high-performance liquid chromatography (HPLC) to determine the position of methyl groups in methylated cytosine. We demonstrated by experimental evidence that M.ApeKI is a DNA (cytosine-5)-methyltransferase and that it recognizes the sequence 5′-GCWGC-3′, the same recognition sequence as the associated endonuclease ApeKI. Furthermore, we discovered that the optimum temperature of M.ApeKI is over 70°C. We suggest that M.ApeKI has strong thermostability, as activity was still observed after incubation at 90°C for 10 h. Therefore, we conclude that *A. pernix* K1 has an R-M system and that M.ApeKI is a thermostable DNA (cytosine-5)-methyltransferase.

## RESULTS

### Phylogenetic tree construction and amino acid alignment.

**FIG 1:**
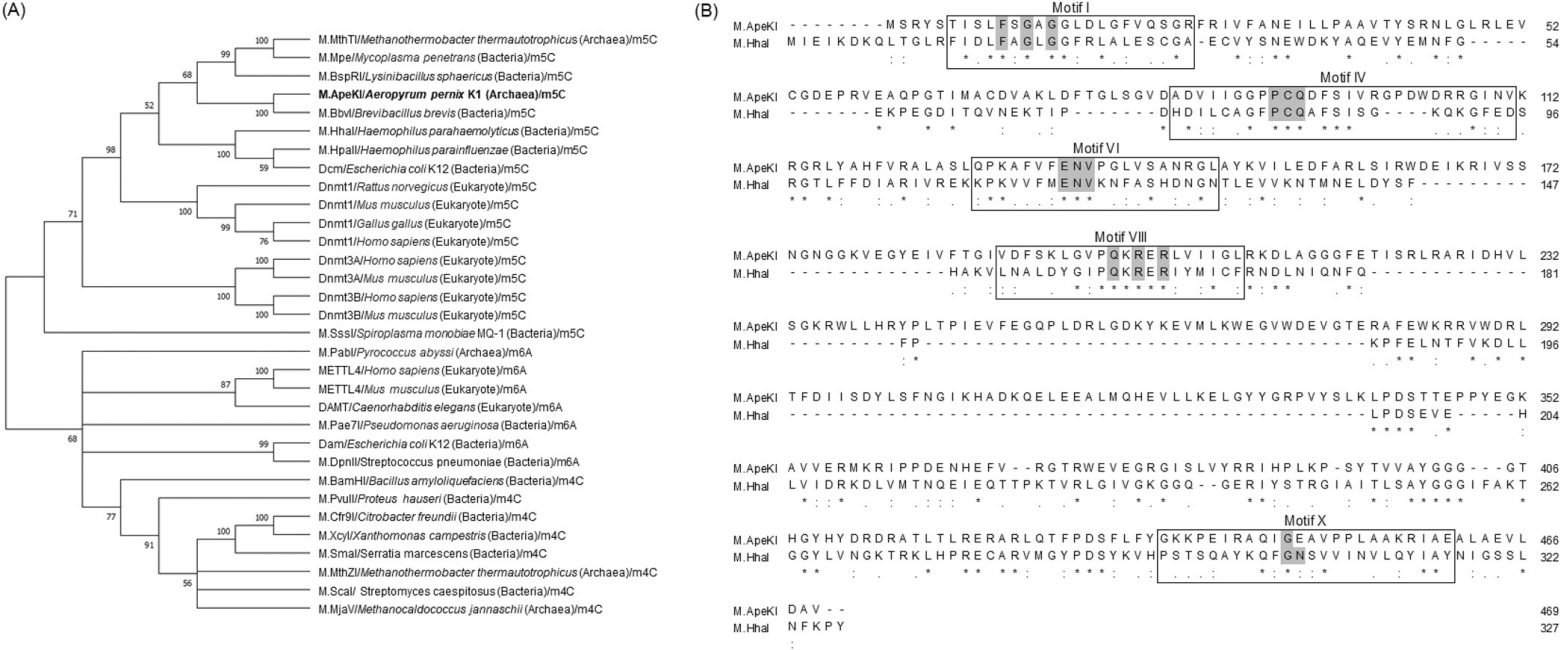
Prediction of modified DNA base by MTases. (A) The evolutionary history was inferred using the neighbor-joining method (34). The optimal tree is shown. The percentage of replicate trees in which the associated taxa clustered together in the bootstrap test (1,000 replicates) is shown next to the branches (35). The evolutionary distances were computed using the p-distance method (36) and are in the units of the number of amino acid differences per site. This analysis involved 32 amino acid sequences. All positions containing gaps and missing data were eliminated (complete deletion option). There were a total of 169 positions in the final data set. Evolutionary analyses were conducted in MEGA X (37). (B) Amino acid alignment. Asterisk (*), perfect alignment; colon (:), a site belonging to a group exhibiting strong similarity; period (.), a site belonging to a group exhibiting weak similarity. For the common motif of DNA (cytosine-5)-methyltransferase, we referred to the study of M.HhaI (28, 29). As a result, in the highly conserved motifs, FXGXGX in motif I, PCQ in motif IV, ENV in motif VI, and QXRXR in motif VIII were observed; however, GN in motif X was not observed.

We investigated whether M.ApeKI is likely to be a DNA (adenine-6)-methyltransferase, DNA (cytosine-5)-methyltransferase, or DNA (cytosine-4)-methyltransferase by phylogenetic tree construction (Fig. 1A) and amino acid alignment. In Fig. 1A, DNA (adenine-6)-methyltransferase, DNA (cytosine-5)-methyltransferase, or DNA (cytosine-4)-methyltransferase were used, and these have been reviewed in UnprotKB/Swiss-Prot. The results indicated that M.ApeKI and the DNA (cytosine-5)-methyltransferase share a recent common ancestor.

Moreover, the amino acid sequence of M.ApeKI was aligned with the known m5C bacterial MTase M.HhaI. Figure 1B shows the results of the amino acid alignment. The alignment of the amino acid sequence was analyzed using multiple sequence comparison by log-expectation (MUSCLE) (26).

### Extraction and purification.

**FIG 2:**
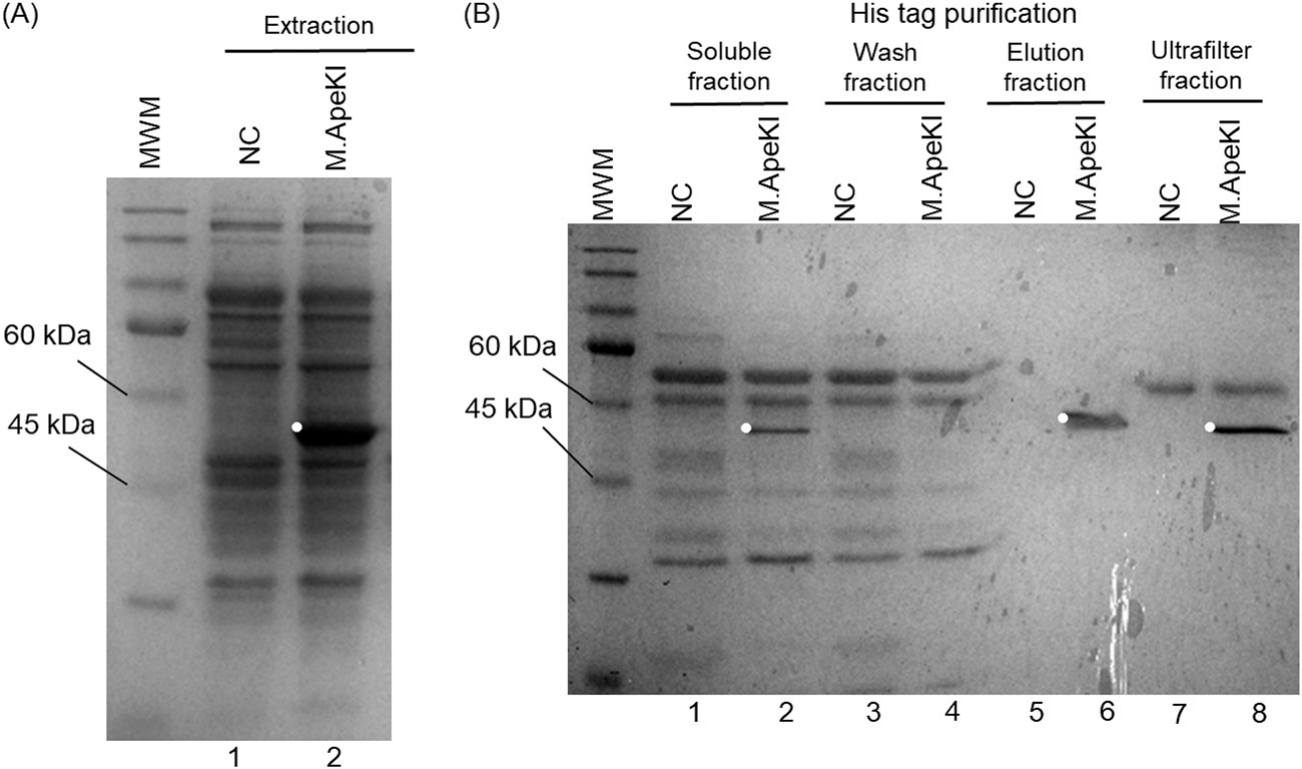
Extraction and purification of M.ApeKI. (A) The results of extraction. The molecular weight marker was protein marker PM2500 from Smobio. Negative control (NC) was not included. (B) Results of purification. The band near to the target molecular weight was observed in the soluble fraction, elution fraction, and ultrafilter fraction in the M.ApeKI lane (white circle). Polyacrylamide gel electrophoresis was performed in 12% separation gel in a buffer containing 25 mM Tris, 0.1% SDS, and 192 mM glycine at 30 mA. It was stained with EzStain AQua from CBB.

Figure 2 shows the results of SDS-PAGE for the extracted and purified M.ApeKI. The estimated molecular weight of M.ApeKI was 56.4 kDa. The negative control (NC) was from E. coli, including the pCold I vector without M.ApeKI, and it was treated in the same way as the M.ApeKI sample. Figure 2A shows the results of the extraction of the NC and M.ApeKI. The band near the estimated molecular weight of M.ApeKI was unobserved in the NC lane. Conversely, in the M.ApeKI lane, a dark band was observed in the estimated molecular weight of M.ApeKI.

Figure 2B shows the results of the purification and ultrafiltration of M.ApeKI. In the soluble fraction, a single band of M.ApeKI was observed near the target molecular weight, but that band was not observed in the NC lane (Fig. 2B, lanes 1 and 2). After the extraction, heat treatment was effective for a shift of M.ApeKI from the insoluble fraction to the soluble fraction. In the wash fraction, the band was unobserved (Fig. 2B, lanes 3 and 4). In the elution fraction, the band that is near the target molecular weight of M.ApeKI was not observed in the NC lane but was observed in the M.ApeKI lane (Fig. 2B, lanes 5 and 6). In addition, in the ultrafiltration fraction, a band was not observed near the target molecular weight of M.ApeKI in the NC lane, but a band was detected over 60 kDa in the M.ApeKI lane (Fig. 2B, lane 7). A single band was detected near the target molecular weight of M.ApeKI in the M.ApeKI lane, and a band was observed over 60 kDa, similar to that in the NC lane.

### Determination of recognition sequence.

**FIG 3:**
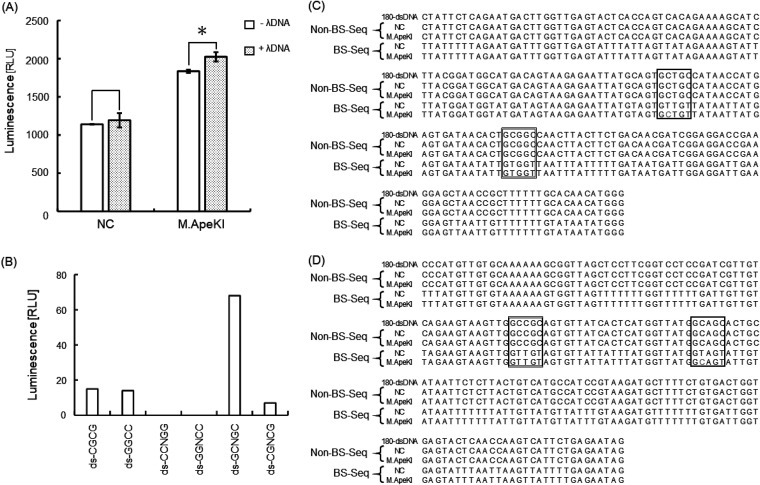
Determination of recognition sequence. (A) Activity of M.ApeKI was measured by MTase-Glo methyltransferase assay. Statistical significance (*P* value) was determined by two-tailed Student’s *t* test (*P* < 0.05). (B) To estimate the recognition sequence of M.ApeKI, MTase-Glo methyltransferase assay was used. Bar chart values indicate correction values, which subtracts −λ DNA from +λ DNA. BS-seq was used for the determination of the recognition sequence of M.ApeKI sense strand (C) and antisense strand (D). “Non-BS-seq” indicates 180-dsDNA not treated with bisulfite, and “BS-seq” indicates 180-dsDNA treated with bisulfite. “NC” indicates 180-dsDNA treated with the negative control, and “M.ApeKI” indicates 180-dsDNA treated with M.ApeKI. The single border indicates the 5′-GCTGC-3′ and 5′-GCAGC-3′ sequences, and the double border indicates 5′-GCGGC-3′ and 5′-GCCGC-3′. Gray letters indicate methylcytosine in 5′-GCTGC-3′ and 5′-GCAGC-3′.

The recognition sequence of M.ApeKI was characterized by methylation activity. Figure 3A shows the results of the confirmation of DNA methylation activity using double-stranded λ DNA as a substrate and a luminescence assay for the conversion of SAM (methyl donor) to its product *S*-adenosylhomocysteine (SAH). The results showed that in M.ApeKI, there was a significant difference between including λ DNA (+λ DNA) and excluding λ DNA (−λ DNA). In the NC, however, there was no significant difference. Thus, M.ApeKI has DNA methylation activity.

Figure 3B shows the results of the characterization of the recognition sequence by methylation activity using double-stranded oligonucleotides. When we used double-stranded GCNGC (ds-GCNGC) as a substrate, we observed the highest activity. The methylation activities of M.ApeKI on the other substrates showed little or no activity. Thus, M.ApeKI has predicted specificity for 5′-GCNGC-3′.

Figure 3C and D show the results of the determination of the base (N) in the 5′-GCNGC-3′ sequence (N = A, T, C, G) that used 180-bp double-stranded DNA (180-dsDNA). In this experiment, methylcytosine was detected by BS-seq. The sequence of the substrate is shown in Table 1. We analyzed the sense strand (Fig. 3C) and the antisense strand (Fig. 3D) in 180-dsDNA. From the results of BS-seq, the second cytosine in 5′-GCTGC-3′ and 5′-GCAGC-3′ sequence was observed to have been modified to methylcytosine. Conversely, no cytosine residues in the 5′-GCGGC-3′ and 5′-GCCGC-3′ sequences were observed to have been methylated.

**TABLE 1:** Primers, double-stranded DNAs, and substrates used in this study

Expt	Name		Sequence (5′ to 3′)	bp	Tm [°C]^*a*^
Constructed plasmid	APE ORF(EcoRI) Fw		GCGGAATTCATGTCACGATATAGTACCATTAGC	33	62
	Dnmt1(APE) PstI+fin Rv		CATCTGCAGTTATCACACAGCATCCAGAACTTC	33	63
Sequence analysis	pCold I_Universal_F		GTAAGGCAAGTCCCTTCAAGAG	22	57.7
	pCold-R Primer		GGCAGGGATCTTAGATTCTG	20	55.4
	Dnmt1(APE)primer walk Fw		AACGGGAACGCCTGGTAATCATCG	24	61
	Dnmt1(APE)primer walk Rv		CCAAGACGATCCAACGGTTGAC	22	60
Determination ofrecognition sequence					
MTase-Glo methyltransferase assay	double-stranded CGCG (ds-CGCG)		CGCGCGCGCGCGCGCG	16	75
	double-stranded GGCC (ds-GGCC)		GGCCGGCCGGCCGGCC	16	72
	double-stranded CCNGG (ds-CCNGG)		CCAGGCCCGGCCGGGCCTGG	20	73
	double-stranded GGNCC (ds-GGNCC)		GGACCGGCCCGGGCCGGTCC	20	73
	double-stranded GCNGC (ds-GCNGC)		GCAGCGCCGCGCGGCGCTGC	20	76
	double-stranded CGNCG (ds-CGNCG)		CGACGCGCCGCGGCGCGTCG	20	75
			
BS-seq	180-bp double-stranded DNA (180-dsDNA)		CTATTCTCAGAATGACTTGGTTGAGTACTCACCAGTCACAGAAAAGCATCTTACGGATGGCATGACAGTAAGAGAATTATGCAGTGCTGCCATAACCATGAGTGATAACACT**GCGGC**CAACTTACTTCTGACAACGATCGGAGGACCGAAGGAGCTAACCGCTTTTTTGCACAACATGGG	180	
	BS-seq Fw		TTATTTTTAGAATGATTTGGTTGAG	25	50
	BS-seq Rv		CCCATATTATACAAAAAAACAATTA	25	48
	Non-Bs-seq Fw		CTATTCTCAGAATGACTTGGTTGAG	25	55
	Non-Bs-seq Rv		CCCATGTTGTGCAAAAAAGCGGTTA	25	61
Determination of modified base	GCWGC Fw	double-stranded GCWGC (ds-GCWGC)	GCAGCGCTGCGCAGCGCTGCGCAGCGCTGCGCAGC	35	83
	GCWGC Rv		GCTGCGCAGCGCTGCGCAGCGCTGCGCAGCGCTGC	35	83

a The temperature of melting (Tm) is defined as the temperature at which one half of the DNA duplex will dissociate to become single stranded and indicates the duplex stability.

### Determination of the modified position in methyl cytosine.

**FIG 4:**
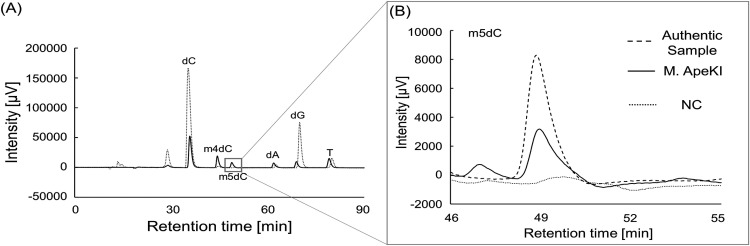
Determination of the modified position of methyl groups in methylcytosine. All nucleotides were detected at 287 nm, the absorption maximum wavelength of m5dC (data not shown). (A) The result of HPLC analysis. Six deoxyribonucleosides (dC, m4dC, m5dC, dA, dG, and T) as authentic sample, the retention times were as follows: dC = 35.8, m4dC = 44.4, m5dC = 48.9, dA = 61.9, dG = 68.9, T = 79.1 min. (B) Magnified view of the retention time of m5dC.

We determined the modified position in methyl cytosine by HPLC. Figure 4A shows the results of the determination of the modified position in methyl cytosine by M.ApeKI, and the magnification view for the retention time of 2′-deoxy-5-methylcytidine (m5dC) is shown in Fig. 4B. Double-stranded GCWGC (ds-GCWGC) was used as the substrate (Table 1) and was detected by HPLC after digestion. Five deoxyribonucleosides (2′-deoxycytidine, dC; 2′-deoxy-4-methylcytidine, m4dC; 2′-deoxyadenosine monohydrate, dA; 2′-deoxyguanosine hydrate, dG; thymidine, T), except m5dC, were detected in the NC (Fig. 4B). The ds-GCWGC was treated with purified M.ApeKI (solid line in Fig. 4A) or M.SssI as the positive control (methylates CpG to m5CpG; data not shown), and six deoxyribonucleosides (dC, m4dC, m5dC, dA, dG, and T) were detected. The peak of m5dC in M.ApeKI was consistent with the peak of m5dC from the authentic sample and from M.SssI, which is known as DNA (cytosine-5)-methyltransferase (27).

### Optimum temperature and thermostability of M.ApeKI.

**FIG 5:**
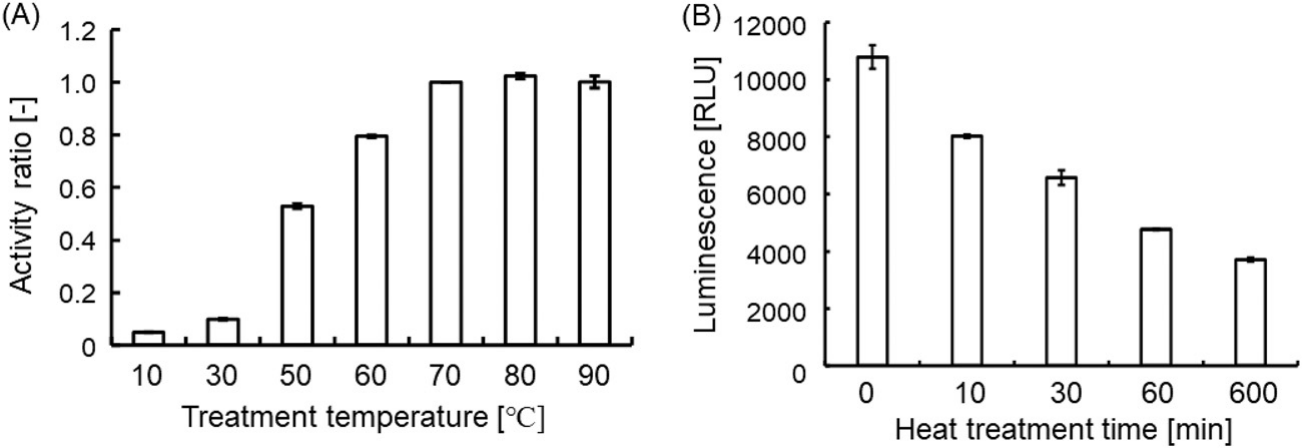
Evaluation of the optimum temperature and investigation of thermostability. The activity of M.ApeKI was measured by MTase-Glo methyltransferase assay. (A) The detected optimum temperature. M.ApeKI was reacted with double-stranded DNA as a substrate. Activity ratio was based on the result of treatment at 70°C. (B) The evaluated thermostability. M.ApeKI was reacted with double-stranded DNA as a substrate (Table 1).

The optimum temperature and thermostability of M.ApeKI were determined by measurement of methylation activity via luminescent assay of the SAM product SAH. In this experiment, ds-GCWGC was used as a substrate (Table 1). The lowest activity was observed at 10°C (Fig. 5A), and at over 70°C, the highest luminescence value was detected. Figure 5B shows the results of the thermostability investigation. M.ApeKI was heated at 90°C for 10 min, 30 min, 60 min, and 10 h before the evaluation. As a result, the activity decreased by 40% when M.ApeKI was heated at 90°C for 30 min, and the activity decreased by 60% when M.ApeKI was heated at 90°C for 60 min. Furthermore, when M.ApeKI was heated at 90°C for 10 h, the activity decreased by 66% compared with that of the unheated sample.

## DISCUSSION

In this study, we characterize the properties of M.ApeKI. The phylogenetic results suggest that M.ApeKI is more closely related to DNA (cytosine-5)-methyltransferases than any other MTase. DNA (cytosine-5)-methyltransferases have 10 conserved motifs, and motifs I, IV, VI, VIII, and X were reported to be especially highly conserved (28, 29). We focused on these motifs. As a result, conservation of amino acid motifs I, IV, VI, and VIII (but not X) was observed. The highly conserved motifs are reported to play significant roles (28, 30) as follows: motifs I and X are involved in SAM binding, motifs IV and VI are involved in catalysis, and motif VIII is involved in protonation of the target cytosine. The motifs involved in catalysis were observed in M.ApeKI; thus, it was predicted to be an active DNA (cytosine-5)-methyltransferase.

To determine the recognition sequence, ds-GCNGC showed the highest methylation activity. Moreover, we performed the determination of the base (N) in 5′-GCNGC-3′ by BS-seq. As a result, methylcytosine was observed in the second cytosines of 5′-GCTGC-3′ and 5′-GCAGC-3′. Conversely, the methylation activities of M.ApeKI were very low when ds-CGCG, ds-GGCC, or ds-CGNCG were used as substrates. However, from the results of BS-seq, a methylcytosine was not observed in 5′-GGCC-3′. Therefore, we suggested that, while 5′-CGCG-3′ and 5′-CGNCG-3′ were not assessed via BS-seq, these sequences were not methylated by M.ApeKI. We concluded that M.ApeKI acts on the sequence 5′-GC(A/T)GC-3′.

We also investigated the position where the methyl group was added to cytosine by M.ApeKI and found that a peak with the same retention time as that of m5dC was observed in the substrate treated with M.ApeKI. A peak was also observed at the same retention time of m5dC from the substrate treated with M.SssI, which is a known DNA (cytosine-5)-methyltransferase (27), and a peak with the same retention time as m5dC was not observed in the NC. A peak with the same retention time as that of m4dC was observed in the substrate treated with M.ApeKI or M.SssI; however, the NC, which was not treated with any enzyme, also showed a peak at m4dC. The intensity ratio of adenine to m4dC was the same as that of the substrate treated with M.ApeKI or M.SssI and the NC, which was not treated with any sample (data not shown). This result suggests that the use of the enzymes was not related to a peak at the same retention time as m4dC. The study of O’Brown et al. showed that enzymes for digesting DNA contain unmethylated DNA and methylated DNA (14). It is interpreted from this result that M.ApeKI is a DNA (cytosine-5)-methyltransferase. In general, thermophilic bacteria that modify cytosines have DNA (cytosine-4)-methyltransferases that allow them to avoid m5C-to-T mutation. Therefore, the details regarding m5C in thermophilic archaea require further elucidation.

The highest activity was observed after treatment with M.ApeKI at over 70°C, and the activities at 70°C, 80°C, and 90°C were not significantly different. However, DNA as a substrate and SAM as a methyl group donor were predicted to be unstable at high temperature (31, 32); thus, we were concerned that the accurate measurement of methylation activity is difficult at high temperature. In this study, we concluded that the optimum temperature of M.ApeKI is 70°C to 90°C. The novel method for the determination of the optimum temperature of thermostable MTase should be refined further. Moreover, the activity of M.ApeKI was still detected when it was heated at 90°C for 10 h, indicating that M.ApeKI is tolerant to high temperatures.

We concluded that M.ApeKI is a thermostable DNA (cytosine-5)-methyltransferase, which adds a methyl group to the second cytosine in 5′-GCWGC-3′. This recognition sequence corresponds to the recognition sequence of ApeKI. Thus, we suggest that *A. pernix* K1 has an R-M system. Further investigation into the construction of M.ApeKI and its functions will help in elucidating the thermostability mechanism.

## MATERIALS AND METHODS

### Materials and chemicals.

For all experiments, primers were purchased from Eurofins Genomics (Ebersberg, Germany). Sequence analysis was performed by Fasmac Co., Ltd. (Kanagawa, Japan) using the MUSCLE from the EMBL-EBI. The known methyltransferase data were from the UniProt database. The sequences for primers and substrates are shown in Table 1. In the substrate of the determination of recognition sequence by BS-seq, the underline shows the 5′-GC(T/A) GC-3′ sequence, and the bold shows the 5′-GC(G/C) GC-3′ sequence. These were predicted to be the recognition sequence of M.ApeKI.

For the construction of the plasmid, the M.ApeKI synthetic sequence was purchased from Eurofins Genomics. The *Ex Taq* hot-start PCR reagent and the restriction enzymes were purchased from TaKaRa Bio Inc. (Shiga, Japan). The vector, pCold I, used in this study was also purchased from TaKaRa. The gel purification system used was a FavorPrep GEL/PCR purification minikit from Favorgen Biotech Corporation (Ping-Tung, Taiwan). Ligation Mighty Mix from TaKaRa was used for ligation. The E. coli strain, JM109, was purchased from Toyobo Co., Ltd. (Osaka, Japan), and Luria-Bertani (LB) medium was purchased from Nacalai Tesque, Inc. (Kyoto, Japan).

The extraction of M.ApeKI was performed using protease inhibitor cocktail for general use (100×) from Nacalai Tesque, Inc., and M.ApeKI was extracted using an ultrasonic disrupter (UD-201; Tomy Digital Biology Co., Ltd., Tokyo, Japan). M.ApeKI was purified using a HisLink spin protein purification system (Promega Corporation, WI, USA), and liquid was replaced using a 3K Omega membrane (Pall Corporation, NY, USA). The electrophoresis reagent gel staining solution used after the SDS-PAGE was EzStain Aqua from ATTO Corporation (Tokyo, Japan). Bisulfite treatment was performed using an EpiTect Fast DNA bisulfite kit from Qiagen (Hilden, Germany), and the sample was amplified using KOD-Multi&Epi from Toyobo.

To measure methylation activity, we used an MTase-Glo methyltransferase assay kit from Promega and a plate reader from Mithras LB940 from Berthold Technologies (Baden-Württemberg, Germany). For the substrate, a double-stranded oligonucleotide was produced using thermal cycler FTGENE-Y2 from Techne (Staffordshire, UK). λ DNA from λ Hind III (TaKaRa) was used as the substrate for the confirmation of methylation activity.

The HPLC system comprised a three-line degasser (JASCO Corporation, Tokyo, Japan), a ternary gradient unit (JASCO), an intelligent column oven (JASCO), an intelligent HPLC insert pump (JASCO), an intelligent UV-visible (UV-Vis) detector and interface box (JASCO), and an InertSustain AQ-C_18_ column (GL Sciences Inc., Tokyo, Japan). dC, dA, T, dG, and m5dC were purchased from Tokyo Chemical Industry (Japan). m4dC was purchased from Biosynth Carbosynth (Berkshire, UK). All samples were filtrated using a syringe-driven filter (0.20 μm) from Merck Millipore (MA, USA) before injection to HPLC. The double-stranded oligonucleotide was digested using Nucleoside Digestion Mix from New England BioLabs Inc. (NEB, MA, USA).

### Phylogenetic tree construction and amino acid alignment.

For this analysis, M.ApeKI was compared with DNA (cytosine-5)-methyltransferase, DNA (cytosine-4)-methyltransferase, and DNA (adenine-6)-methyltransferase. Table 2 shows the MTases list for the construction of a phylogenetic tree. The phylogenetic tree (Fig. 1A) was constructed using MEGA-X software and the neighbor-joining method. For alignment analysis, MUSCLE was used.

**TABLE 2:** DNA methyltransferases used in this phylogenetic tree

Type or no.	Accession no. in Uniprot	Protein name	Scientific name	Organism
DNA (cytosine-5)-methyltransferase				
1	P13906	M.BspRI	Lysinibacillus sphaericus	Bacteria
2	P05102	M.HhaI	Haemophilus parahaemolyticus	
3	P0AED9	Dcm	Escherichia coli K-12	
4	P15446	M.HpaII	Haemophilus parainfluenzae	
5	P15840	M.SssI	Spiroplasma monobiae MQ-1	
6	Q8EVR5	M.Mpe	Mycoplasma penetrans	
7	P34905	M.BbvI	Brevibacillus brevis	
8	P29567	M.MthTI	Methanothermobacter thermautotrophicus	Archaea
9	Q92072	Dnmt1	Gallus gallus	Eukaryote
10	P26358	Dnmt1	Homo sapiens	
11	Q9Z330	Dnmt1	Rattus norvegicus	
12	P13864	Dnmt1	Mus musculus	
13	Q9Y6K1	Dnmt3A	Homo sapiens	
14	O88508	Dnmt3A	Mus musculus	
15	Q9UBC3	Dnmt3B	Homo sapiens	
16	O88509	Dnmt3B	Mus musculus	
DNA (adenine-6)-methyltransferase				
1	P0AEE8	Dam	Escherichia coli K-12	Bacteria
2	P04043	M.DpnII	Streptococcus pneumoniae	
3	P05103	M.Pae7I	Pseudomonas aeruginosa	
4	Q9V2B5	M.PabI	*Pyrococcus abyssi*	Archaea
5	Q09956	DAMT	Caenorhabditis elegans	Eukaryote
6	Q8N3J2	METTL4	Homo sapiens	
7	Q3U034	METTL4	Mus musculus	
DNA (cytosine-4)-methyltransferase				
1	P11409	M.PvuII	Proteus hauseri	Bacteria
2	P23941	M.BamHI	Bacillus amyloliquefaciens	
3	P14243	M.Cfr9I	Citrobacter freundii	
4	P14230	M.SmaI	Serratia marcescens	
5	O52692	M.ScaI	*Streptomyces caespitosus*	
6	P30774	M.XcyI	Xanthomonas campestris pv. *cyanopsidis*	
7	Q58893	M.MjaV	Methanocaldococcus jannaschii	Archaea
8	P29568	M.MthZI	Methanothermobacter thermautotrophicus	

### Construction of the plasmid.

The vector was constructed for the expression of M.ApeKI in E. coli. M.ApeKI was amplified by PCR using *Ex Taq* HS with a template based on the synthesized plasmid. The conditions of PCR were as follows: 98°C for 30 s, 98°C for 10 s, 58°C for 30 s, 72°C for 90 s, and 72°C for 90 s. Then, the sample was held at 4°C. The two primers that were used are the EcoRI site before the M.ApeKI sequence, and the other is the M.ApeKI sequence in addition to the stop codon and the PstI site (shown in Table 1).

After amplification, the PCR product was gel purified. The pCold I vector and the purified PCR product were reacted at 37°C for 2 h with PstI and EcoRI. After the reaction, the product was gel purified, and then, M.ApeKI was introduced into pCold I.

The recombinant plasmid was transformed into E. coli JM109 by heat shock treatment. The transformed E. coli was cultured at 37°C in 50 ml of LB medium with ampicillin. The plasmid was extracted from the cultured E. coli by the alkaline lysis method. The extracted plasmid was gel purified, and the insert sequence was analyzed. The primers used for sequence analysis are shown in Table 1.

### Extraction and purification of M.ApeKI.

M.ApeKI was extracted and purified for the evaluation of its properties. The transformed E. coli was cultured at 37°C overnight in 100 ml of LB medium, including ampicillin as a preculture. Then, it was cultured in water at 15°C for 30 min and incubated at 15°C for 24 h after the addition of isopropyl-β-d-thiogalactopyranoside (IPTG) to a final concentration of 0.1 mM. All of the culture medium in the 50-ml tube was centrifuged at 7,000 × *g* for 5 min at 4°C. The supernatant was discarded, and cells were washed in 0.2 M phosphoric acid buffer (pH 7.4). Cells were suspended in 2 ml of 0.2 M phosphoric acid buffer and protease inhibitor cocktail for general use (100×) and were extracted using an ultrasonic disrupter. The extract was placed in a 1.5-ml tube, was heated at 90°C for 10 min, and then centrifuged at 9,400 × *g* for 30 min at 4°C. The supernatant was regarded as a soluble fraction and was purified. The elution buffer was replaced with 1× reaction buffer (Promega; no. TM453) by ultrafiltration. In this study, M.ApeKI as used for evaluation contains 15 amino acids including a His tag at the N terminus.

### Measurement of methylation activity.

The recognition sequence and optimum temperature were determined by measurement of methylation activity. For the measurement of the DNA methylation activity of M.ApeKI, 0.39 mmol of substrate, 19 μg of the purified M.ApeKI, 32 nmol of SAM, and 0.1 mg/ml of bovine serum albumin (BSA) were mixed. The reactant was evaluated using the activity, and the level of luminescence of the reaction solution was measured by using a plate reader. A double-stranded oligonucleotide as a substrate was produced using a thermal cycler that contained 45 μM PCReady primer and annealing buffer (1 mM Tris-HCl, 100 mM NaCl, and 0.1 mM EDTA). The conditions after incubation were for 5 min at 95°C, and then the temperature was decreased by 1°C/min for 25 min. To confirm the methylation activity of M.ApeKI, λ HindIII digest was used, and the reactant was incubated at 40°C for 2 h. To estimate the optimum temperature of M.ApeKI, ds-GCWGC was used as the substrate, and the reactant was incubated at 40°C for 2 h. To evaluate the optimum temperature of M.ApeKI, the reactant was incubated at 10°C, 30°C, 50°C, 60°C, 70°C, 80°C, and 90°C for 2 h.

### Determination of the recognition sequence of M.ApeKI.

The recognition sequence of M.ApeKI was determined by BS-seq. The conditions of treatment were the same as those for the measurement of methylation activity. The substrate used was an amplicon that included a part of the β-lactamase sequence, and the reactant was incubated at 40°C for 24 h. The bisulfite treatment was performed using an EpiTect Fast DNA bisulfite kit. After the treatment, the sample was amplified by PCR using a KOD Multi & Epi, and the primers are shown in Table 1. The conditions for PCR were 40 cycles at 94°C for 2 min, 98°C for 10 s, 53°C for 30 s, and 68°C for 15 s, and then, the sample was held at 4°C. After gel purification, alignment analysis was performed.

### HPLC.

The modified positions of cytosine by M.ApeKI were determined by HPLC. For the conditions for HPLC, we referred to a previous report (33). This experiment used 0.1% formic acid in Milli-Q and 0.1% formic acid in methanol. The HPLC program was gradient elution. The 0.1% formic acid in methanol was increased to 0.15 ml/min from 0 to 22.5%, and 20 μl of the sample was injected.

dC, dA, T, m5dC, and m4dC were dissolved in Milli-Q water, and dG was dissolved in methanol. After dissolution, all authentic samples were filtrated using a syringe-driven filter (0.20 μm).

A ds-GCWGC as a substrate was produced using a thermal cycler that contained 23 μM PCReady primer of each (forward and reverse) and annealing buffer (1 mM Tris-HCl, 100 mM NaCl, and 0.1 mM EDTA). After incubating for 5 min at 95°C, the temperature was decreased by 1°C/min to 25°C. The sequence of ds-GCWGC is shown in Table 1.

A 0.39-mmol aliquot of ds-GCWGC, 19 μg of the purified M.ApeKI, 32 nmol of AdoMet, and 0.1 mg/ml of BSA were mixed and then reacted for 22 h at 40°C. The sample was digested from the double-stranded oligonucleotide to nucleoside overnight at 37°C using Nucleoside Digestion Mix and was then filtrated using a syringe-driven filter (0.20 μm).
